# Architecting a Bias‐Free Photoelectrochemical CO_2_ Reduction System for Sustainable Formic Acid

**DOI:** 10.1002/advs.202415774

**Published:** 2025-04-04

**Authors:** Yinchao Yao, Zilong Wu, Zhiwei Zhao, Zhiyi Sun, Tiesong Li, Zebiao Li, Xinxin Lu, Zhuo Chen

**Affiliations:** ^1^ Energy & Catalysis Center School of Materials Science and Engineering Beijing Institute of Technology 5 Zhongguancun South Street, Haidian District Beijing 100081 P. R. China; ^2^ China Academy of Information and Communications Technology No. 40, Huayuan Road, Haidian District Beijing 100190 P. R. China; ^3^ PetroChina Shenzhen New Energy Research Institute Co., Ltd Nanshan District Shen Zhen 518052 P. R. China

**Keywords:** bias‐free, CO_2_ reduction, formate, photoelectrochemical

## Abstract

Solar‐driven photoelectrochemical CO_2_ reduction represents a promising approach for the production of renewable liquid fuel but is limited by low photocurrent, the need for an external bias, and low carbon efficiency. This work employs a TiO_2_‐CdS/Se‐ZnSe/S photoanode to drive the sulfur oxidation reaction, achieving a photocurrent density of 12.7 mAcm^−2^ under AM 1.5G illumination and with an 87% retention after 100 h of continuous operation. Furthermore, through tailoring the adsorption capability for the ^*^OCHO intermediate, the Cu_6_Sn_5_ catalyst exhibits a Faradaic efficiency of 92.8% for formic acid at −1.15 V in acidic media and maintains stability above 90% during a 120‐h test. Finally, the constructed system achieves bias‐free photoelectrochemical CO_2_ reduction to HCOOH and delivers a yield of up to 172.9 µmolh^−1^cm^−2^ over an 85‐h long‐term test, outperforming conventional solar‐driven systems. These findings highlight a cost‐effective strategy for solar‐driven liquid fuel production and provide valuable design concepts and insights into the development of photoelectrochemical systems.

## Graphical abstract

A low‐cost, bias‐free photoelectrochemical system for CO_2_ reduction to formic acid (FA) is constructed via a TiO_2_‐CdS/Se‐ZnSe/S photoanode in alkaline solution and Cu_6_Sn_5_ catalyst in acidic electrolyte, in which replacing OER with SOR significantly improve the reaction kinetics. The unique pH‐decoupled system produces 172.9 µmol h^−1^ cm^−2^ FA over 85 h, outperforming traditional solar‐driven systems.

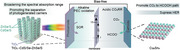

## 1 Introduction

Formic acid (HCOOH), also known as formate, has attracted significant attention due to its exceptional potential in liquid hydrogen storage and transport. As a safe liquid hydrogen carrier, formic acid offers a high hydrogen content (53 g H_2_ L^−1^), substantial hydrogen mass fraction (4.4 wt.%), high density (1.22 g cm^−3^), and spontaneous release of hydrogen, making it highly promising for development and application.^[^ ^1^, ^2^ ^]^ Currently, most research efforts are focused on electrocatalytic CO_2_ reduction and many breakthroughs have indeed been made.^[^ ^3^, ^4^, ^5^, ^6^, ^7^, ^8^, ^9^, ^10^, ^11^ ^]^ In comparison, solar‐driven photoelectrochemical CO_2_ reduction (PEC‐CO_2_RR) can directly utilize solar energy to convert greenhouse gases into high‐value chemicals, making the process more environmentally sustainable. Thus, solar‐driven photoelectrochemistry represents a more promising technological pathway for the production of renewable formic acid.^[^ ^12^, ^13^, ^14^, ^15^ ^]^

To fulfill the high‐efficiency conversion of solar‐driven CO_2_ to liquid fuel, rational design and construction of PEC‐CO_2_RR systems are critical owing to their involvement in various aspects such as semiconductors/catalysts, electrolytes, electrode configurations, etc. From a material perspective, it is necessary to achieve high photocurrent density and sufficient power output, which not only require semiconductors to have broad and strong optical absorption, highly efficient generation and separation of photoexcited carriers but also catalysts to have an excellent catalytic activity to accelerate surface reactions.^[^ ^16^ ^]^ However, the large proportion of the reported photocurrent density is below 1 mA cm^−2[^ ^17^, ^18^, ^19^, ^20^, ^21^, ^22^, ^23^ ^]^ and rarely exceeds 5 mA cm^−2^,^[^ ^12^, ^24^, ^25^, ^26^ ^]^ and the output power is not enough to drive the reactions.^[^ ^12^, ^18^, ^26^, ^27^, ^28^, ^29^ ^]^ Usually, additional bias voltage in the range of 0.4–2 V is required to assist the reactions.^[^ ^30^, ^31^ ^]^ It can be attributed to the following two reasons: limited photovoltage generated by semiconductors and the sluggish oxygen evolution reaction (OER) with a high overpotential.^[^ ^32^, ^33^ ^]^ From a practical application perspective, a photocurrent density of over 10 mA cm^−2^ without an external bias is highly desirable.^[^ ^34^, ^35^ ^]^ Additionally, most PEC‐CO_2_RR systems have been developed in neutral environments, such as KHCO_3_ solutions, which suffer from significant carbon loss as input CO_2_ reacts with OH^−^ to form carbonate ions, which then shuttle to the anode compartment.^[^ ^36^ ^]^ Conducting CO_2_RR in acidic conditions (pH < 3.75) could address this issue, as CO_3_^2^^−^ or HCO_3_^−^ can be reconverted to CO_2_ at the electrode surface.^[^ ^37^, ^38^ ^]^ Pairing acidic CO_2_RR with an alkaline anode cell offers a promising strategy for bias‐free PEC‐CO_2_RR by exploiting the chemical potential difference between the two cells to compensate for insufficient output power generated by semiconductors. However, the high proton concentration in acidic media favors the hydrogen evolution reaction (HER),^[^ ^39^ ^]^ making it difficult to gain a satisfactory Faradaic efficiency (FE) of formic acid. Thus, suppressing HER is crucial for effective PEC‐CO_2_RR in acidic conditions. As for the configuration of photoelectrodes, there are mainly three types: i). photocathode and photoanode; ii). photocathode coupled with electrocatalyst and electroanode; iii). photoanode and electrocathode. The third configuration provides a convenient way to optimize the photoelectrode and electrocatalyst respectively, thus avoiding parasitic light absorption of catalyst and interface recombination between photoelectrode and catalyst.^[^ ^40^, ^41^ ^]^ Therefore, as mentioned above, systematic design, from materials to device level, is essential to develop an effective PEC‐CO_2_RR assembly to accomplish high solar utilization and CO_2_ conversion.

**Figure 1:**
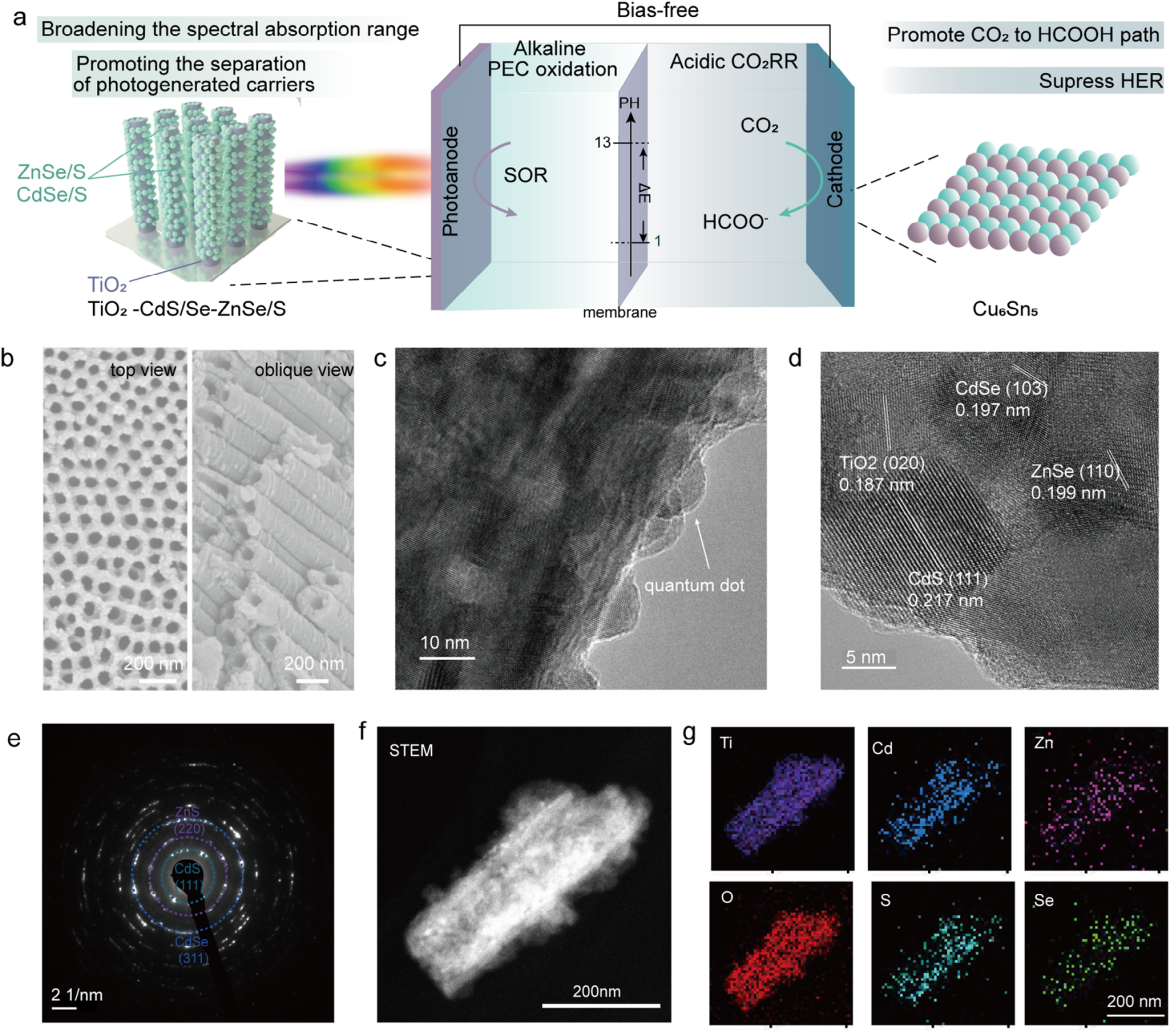
PEC‐CO_2_RR system and characterizations of TCZ photoanode. a) Schematic diagram of solar‐driven bias‐free PEC‐CO_2_RR system for formic acid production. b) SEM, c) TEM, d) HRTEM, e) SEAD, f) STEM images, and g) EDS mapping results of TCZ photoanode.

In this work, we implement a bias‐free PEC‐CO_2_RR setup using TiO_2_‐CdS/Se‐ZnSe/S (TCZ) photoanode in alkaline solution and Cu_6_Sn_5_ electrocathode in acidic electrolyte to achieve solar‐driven conversion from CO_2_ to HCOOH (**Figure** **1**a), where the OER at anode is replaced by the sulfur oxidation reaction (SOR, E° = 0.48 V). By assembling narrowband quantum dots on TiO_2_ nanotube arrays to form a ladder band structure, the photoanode exhibits wide absorption, efficient photocarrier separation, and reduced interfacial charge transfer resistance, achieving an improved saturated photocurrent density of 12.7 mA cm^−2^ under AM 1.5G illumination. After 100 h of continuous operation, the composite photoanode retains 87% of its initial photocurrent density, demonstrating excellent stability. Subsequently, we optimize the composition of CuSn alloy to regulate the ^*^OCHO affinity, promoting the formic acid pathway in acidic CO_2_RR while effectively suppressing HER. Under strongly acidic conditions with pH 1, the Cu_6_Sn_5_ catalyst exhibits a formic acid FE of 92.8 ± 1.3% at −1.15 V and maintains above 90% FE throughout a 120‐h stability test. In situ infrared spectroscopy results indicate that the optimal selectivity of the Cu_6_Sn_5_ catalyst is due to the strongest adsorption capability for the ^*^OCHO intermediate. Through integrating the above photoanode and electrocatalyst into a pH decoupled system, the PEC‐CO_2_RR setup without an external bias is constructed for efficient solar‐driven formate production, delivering a yield of up to 172.9 µmol h^−1^ cm^−2^ over an 85‐h long‐term test.

## 2 Results and Discussion

### 2.1 Photoanode Design and SOR Performance

For photoelectrodes, developing low‐cost strategies to simultaneously achieve high photocurrent density and photovoltage output is essential. However, most reported photoelectrodes based on low‐cost solution methods usually suffer from limited light absorption and severe charge recombination, which restrict their performance. Although multijunction photoelectrodes via traditional semiconductor processes can improve the performance of photocurrent and photovoltage, these methods significantly increase the cost of materials and fabrication processes. Therefore, we employ a low‐cost solution method to orderly assemble TCZ photoanode for PEC applications (Figure 1a), based on the band structure design (Figure S1, Supporting Information). The preparation of the photoanode involves a two‐step process. Initially, TiO_2_ nanotube arrays (NATs) were fabricated via anodic oxidation, followed by sensitization with CdS/Se‐ZnSe/S quantum dots through successive ionic layer adsorption and reaction. Finally, the nanotube arrays underwent annealing to yield the TCZ sample. The TiO_2_‐CdS/Se, denoted as TC was also prepared (details in the Experimental Section). As shown in Figure 1b and Figure S2 (Supporting Information), scanning electron microscope (SEM) images reveal that the synthesized TiO_2_ NATs exhibit a uniform diameter distribution, predominantly in the 82–92 nm range. X‐ray diffraction (XRD) patterns confirm the presence of the anatase phase (JCPDS No.21–1272). Transmission electron microscopy (TEM) and high‐resolution TEM (HRTEM) images of TCZ (Figure 1c,d) show a uniform distribution of granular quantum dots on the TiO_2_ NATs. Additionally, selected area electron diffraction (SAED) and elemental mapping confirm the presence of CdS, CdSe, ZnSe, and ZnS quantum dots, while energy‐dispersive X‐ray spectroscopy (EDS) maps demonstrate the homogeneous distribution of Ti, O, Cd, Zn, S, and Se elements across the nanotubes (Figure 1e–g). X‐ray photoelectron spectroscopy (XPS) analyses further validate the chemical composition of the TCZ, verifying the presence of Ti2p, O1s, Cd2p, S2p, and Zn2p signals (Figure S4, Supporting Information).

**Figure 2:**
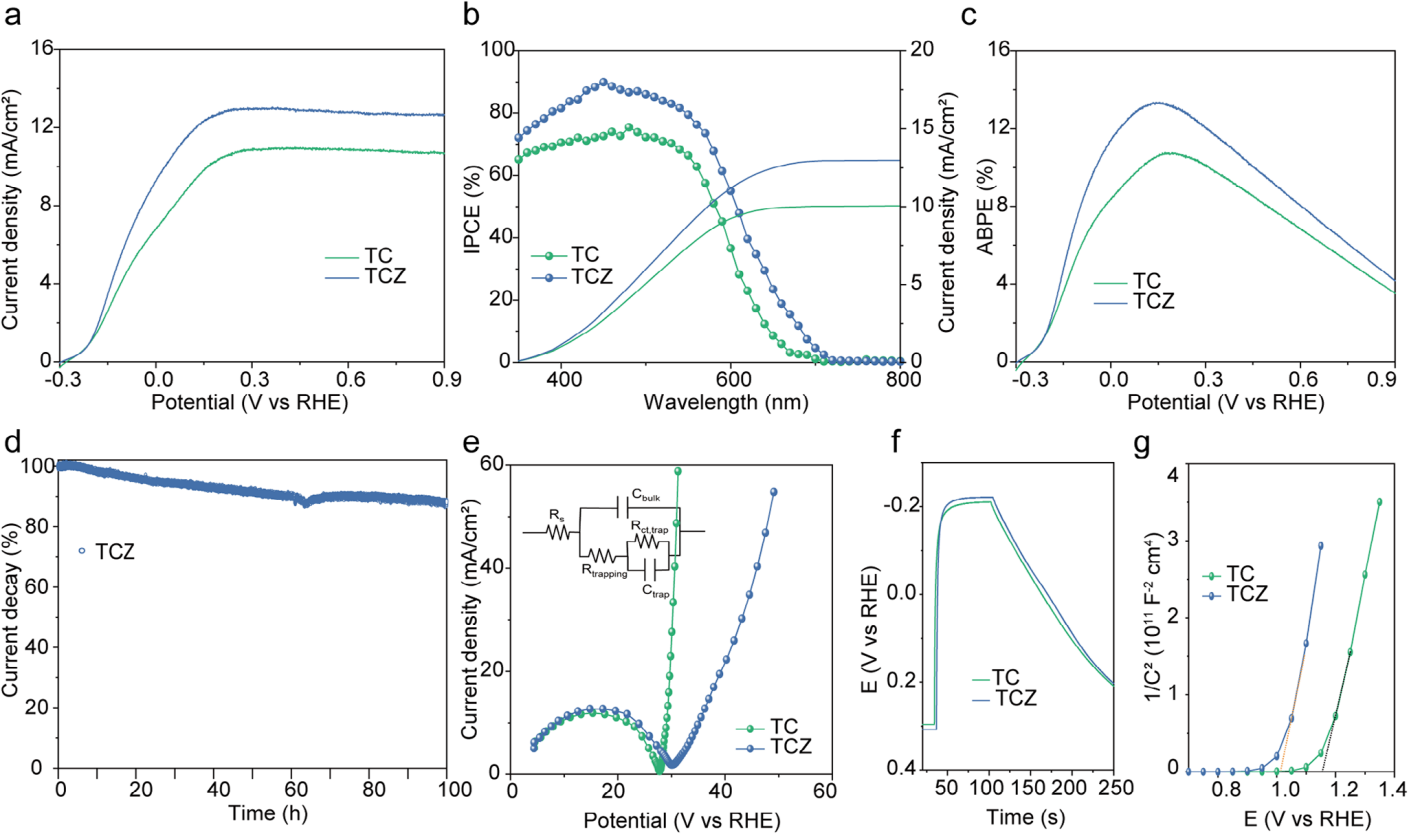
Photoanodic sulfur oxidation. a) LSV of TCZ and TC. b) IPCE, c) ABPE, d) long‐time stability test results of TCZ at 0.3 V. e) electrochemical impedance plots and (inset) equivalent circuit diagrams, f) light‐dark current density variation plots, and g) Mott–Schottky plots for TCZ and TC.

The photoanode samples were tested under AM 1.5G irradiation at an intensity of 100 mW cm^−2^ in a three‐electrode system. As shown in **Figure** **2**a, the saturated current density of TCZ reaches 12.7 mA cm^−2^, showing significant improvement compared to that of TC (10.7 mA cm^−2^). This improvement can be attributed to the introduction of ZnSe and ZnS layers. Due to a higher valence band edge and lower conduction band compared to CdSe, ZnSe can form a gradual band structure with TiO_2_, enhancing light absorption and facilitating photocarrier separation and transport, consistent with the incident photon‐to‐current efficiency (IPCE) results shown in Figure 2b. Additionally, the applied bias photon‐to‐current efficiency (ABPE) results (Figure 2c) demonstrate that the multilayer quantum dots effectively enhance the photoelectric conversion efficiency of TiO_2_. Stability tests on the photoanode samples indicate that after 5 h of continuous operation under illumination at 0.3 V, the current retention of the TCZ sample improves by more than 30% compared to the TC sample (Figure S5, Supporting Information). Furthermore, in a long‐term stability test of 100 h (Figure 2d), the final current of the TCZ photoanode sample remains at 87% of its initial value, showing only a 13% degradation, highlighting its excellent long‐term stability. Moreover, the TCZ sample exhibits lower interfacial charge transfer resistance, a longer lifetime of the photoelectrons, and higher carrier concentration (Table S1, Supporting Information) compared to the TC sample as evidenced by electrochemical impedance spectroscopy (Figure 2e), open‐circuit potential transient decay (Figure 2f) and Mott–Schottky measurements (Figure 2g). The ZnSe/ZnS passivation layer not only broadens the light absorption of the TCZ photoelectrode but also protects the oxidation‐prone CdS/CdSe quantum dots, thereby enhancing the overall performance and durability of the photoanode (Table S1, Supporting Information).

### 2.2 Optimization of CuSn Electrocatalysts and CO_2_RR Performance

**Figure 3:**
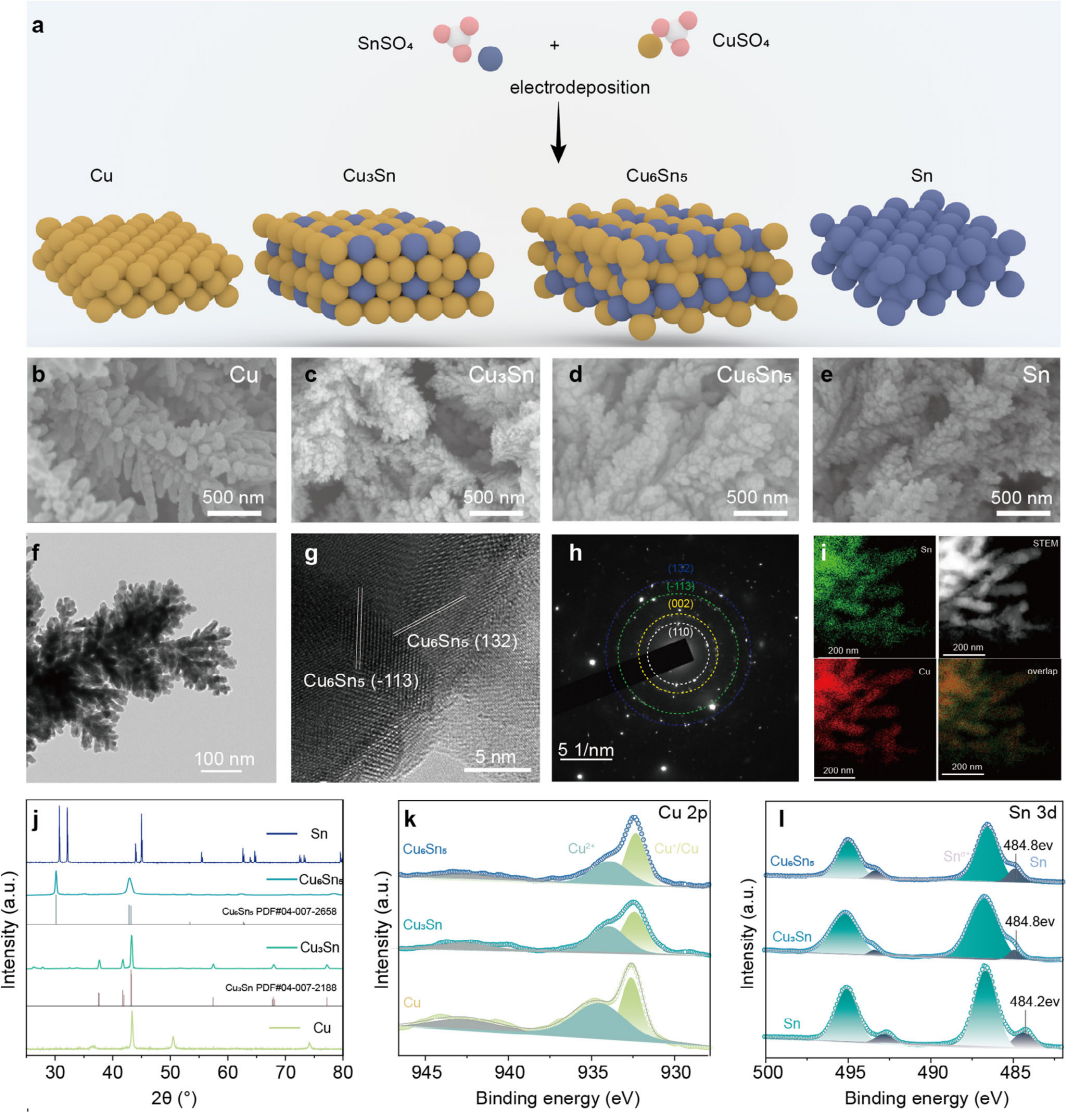
Construction as well as structural characterization of Cu─Sn alloys. a), Schematic of the synthesis of CuSn alloy catalysts. b) SEM images of Cu, c) Cu_3_Sn, d) Cu_6_Sn_5_, e) Sn. f) TEM image, g) HRTEM image, h) SEAD, and i) EDS results of Cu_6_Sn_5_. j) XRD pattern of Cu, Cu_3_Sn, Cu_6_Sn_5_ and Sn, and their k) Cu 2p and l) Sn 3d spectra.

To actualize efficient PEC‐CO_2_RR, high formic acid selectivity is one of the necessary prerequisites. The key to achieving high formic acid selectivity for electrocatalysts lies in the regulation of adsorption capability for the ^*^OCHO intermediate.^[^ ^42^, ^43^, ^44^ ^]^ Therefore, we selected CuSn alloy as an electrocatalyst and optimized its composition to adjust its ^*^OCHO affinity, thereby tailoring the selectivity of formate. As shown in **Figure** **3**a, we synthesized four types of catalysts via varying the deposition currents and precursor solutions (details can be found in the Experimental Section, Supporting Information), including Cu, Cu_3_Sn, Cu_6_Sn_5,_ and Sn catalysts. SEM and TEM reveal that the CuSn alloy samples and the individual Cu and Sn samples prepared by co‐deposition exhibit dendritic and hierarchical structures, which provide abundant surface sites (Figure 3b–f). HRTEM images and SAED patterns of Cu_6_Sn_5_ show interplanar spacings of 0.296 and 0.210 nm, corresponding to the (−113) and (132) crystal planes of Cu_6_Sn_5_, respectively (Figure 3g,h). Additionally, EDS results confirm the uniform distribution of Cu and Sn elements within the Cu_6_Sn_5_ alloy (Figure 3i). XRD patterns further validate the crystalline structure of the Cu_6_Sn_5_ alloy, matching the powder diffraction file (PDF) 04‐007‐2658, with diffraction peaks at 30.1° and 43° corresponding to the (−113) and (132) planes, respectively (Figure 3j), along with the crystalline structure of the Cu_3_Sn alloy (PDF#04‐007‐2188). XPS analysis was employed to investigate the chemical states of Cu, Sn, and the charge transfer between them. The Cu 2p spectra (Figure 3k) indicate that both Cu and CuSn alloys mainly consist of Cu^0^ (932.8 eV) and minor Cu^2+^ (934.5 eV).^[^ ^45^, ^46^ ^]^ Moreover, compared to pure Sn, the Sn^0^ 3d peaks in the CuSn alloys slightly shift to higher binding energies (484.2–484.8 eV) (Figure 3l; Figure S6a, Supporting Information),^[^ ^46^, ^47^ ^]^ suggesting that alloying Cu and Sn results in charge redistribution from Sn to Cu sites. This was further confirmed by the Bader charge analysis results (Figure S6c,d, Supporting Information).

**Figure 4:**
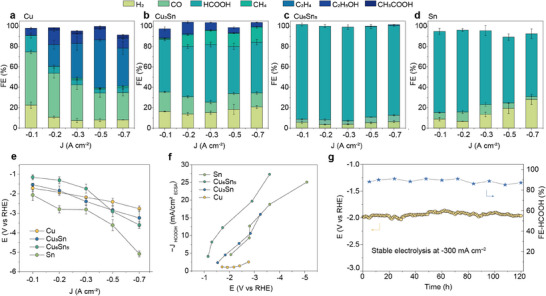
Electrochemical properties of CO_2_RR in acidic medium. FE values of CO_2_RR products of a) Cu, b) Cu_3_Sn, c) Cu_6_Sn_5_, d) Sn catalysts at different current densities, along with their e) current densities and f) normalized formic acid partial currents as a function of the applied potential. Error bars represent standard deviations from three replicate experiments. g) The stability test results of Cu_6_Sn_5_ in a flow cell for over 120 h at a constant current density of −300 mA cm^−2^ demonstrate sustained performance, with minimal degradation observed throughout the duration of the test.

To enhance the carbon efficiency and utilize the chemical potential difference between the anodic cell and the cathodic cell, we evaluate the CO_2_RR performance of these catalysts in acidic media. Specifically, we conducted electrocatalytic tests on Cu, Cu_3_Sn, Cu_6_Sn_5_, and Sn using a gas diffusion electrode as the working electrode and an acidic solution of 0.05 m H_2_SO_4_ and 0.5 m K_2_SO_4_ as the electrolyte in a three‐electrode flow cell. The quantitative analysis of gaseous and liquid products was performed using gas chromatography and nuclear magnetic resonance spectroscopy, respectively. As shown in **Figure** **4**a, the pure Cu catalyst exhibits a broad product distribution, with an FE for formic acid significantly lower than that of other products. In contrast, the CuSn catalysts effectively promote the pathway for CO_2_ to formic acid while suppressing other catalytic pathways. Specifically, within the tested current density range, Cu_3_Sn demonstrates an optimal formic acid FE of 56.4 ± 2.5%, with minor amounts of CO, CH_4_, C_2_H_5_OH, and other by‐products (Figure 4b). As the Sn content increases, Cu_6_Sn_5_ displays the highest formic acid selectivity, reaching a formic acid FE of 92.8 ± 1.3% at −1.15 V (Figure 4c,e). Notably, Cu_6_Sn_5_ maintains an FE of formic acid above 90% over a broad current density range from −0.1 to −0.5 A cm^−2^, significantly exceeding pure Sn (Figure 4d). Furthermore, previous reports have shown that alloying Cu with Sn can weaken the hydrogen adsorption energy on Cu catalysts, thereby suppressing the HER.^[^ ^4^, ^46^, ^48^ ^]^ As demonstrated in Figure 4c, the Cu_6_Sn_5_ catalyst exhibits the best CO_2_RR performance and a significantly lower H_2_ FE compared to the other three catalysts, with a minimum value of 3.53% at a current density of −200 mA cm^−2^. To further analyze the intrinsic catalytic activity, we normalized the partial current for formic acid by the electrochemically active surface area (ECSA) (Figure S7, Supporting Information). The normalized partial current for formic acid on Cu_6_Sn_5_ reaches a maximum of 27.3 mA cm^−2^, demonstrating the highest intrinsic activity for formic acid production (Figure 4f). Moreover, in a flow cell reactor, the Cu_6_Sn_5_ catalyst shows excellent long‐term stability, maintaining an average formic acid FE of ≈90% over 120 h at a reaction rate of −0.3 A cm^−2^ (Figure 4g). Moreover, no significant structural changes or metal leaching were observed, further highlighting the superior stability of the Cu_6_Sn_5_ catalyst (Figure S8 and Table S2, Supporting Information).

### 2.3 In Situ Infrared Mechanistic Studies

**Figure 5:**
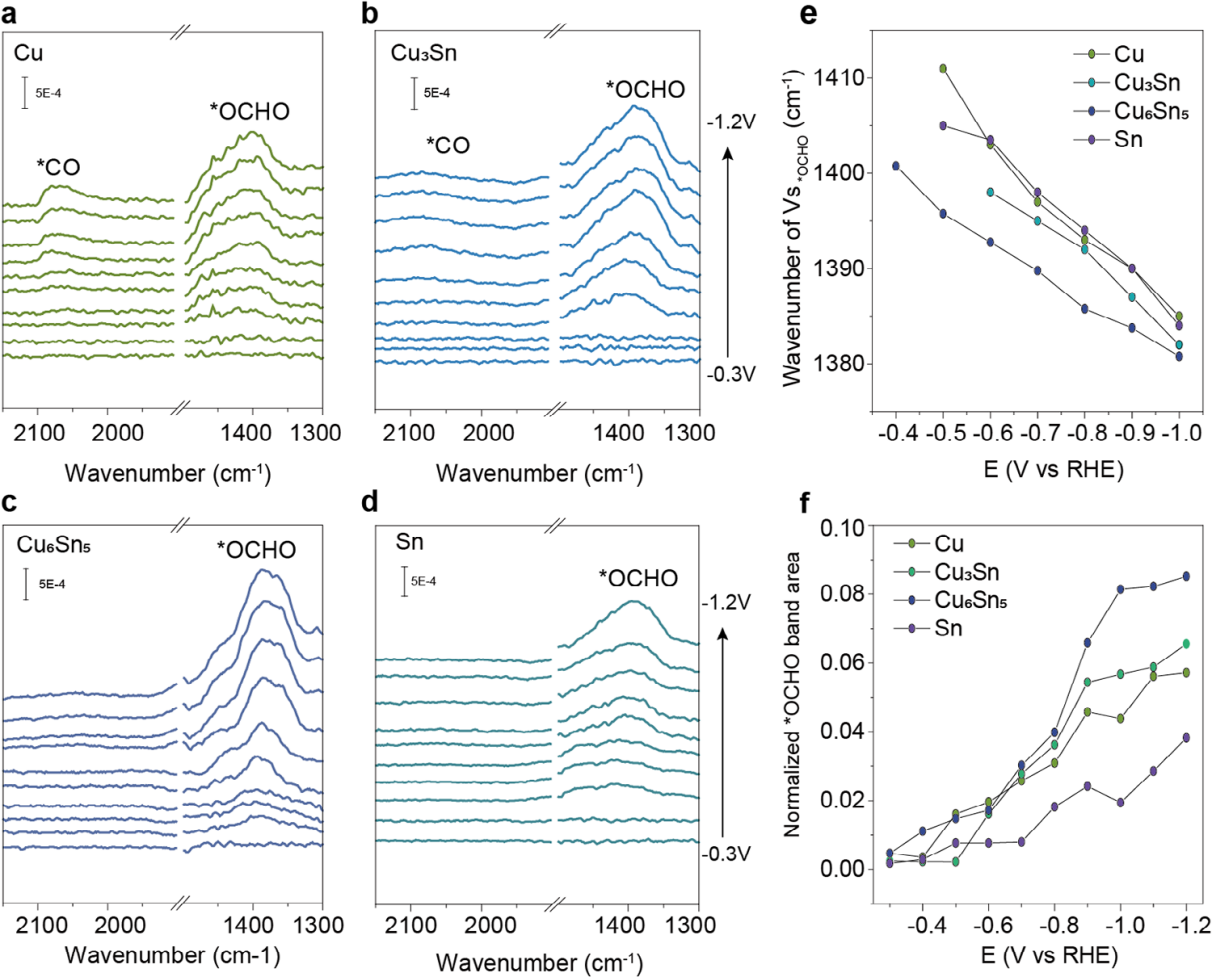
Results of in situ ATR‐SEIRS experiments. ATR‐SEIR spectra of a) Cu, b) Cu_3_Sn, c) Cu_6_Sn_5_, and d) Sn catalysts collected in 0.05 m H_2_SO_4_ + 0.5 m K_2_SO_4_ electrolyte at different potentials. e) Relationship between the vibrational frequencies of ^*^OCHO absorption peaks and the applied potentials. f) Normalized peak area of the ^*^OCHO absorption peaks as a function of the applied potentials, with normalization performed against the largest ν(O─H) band of ^*^H_2_O.

To investigate the effect of alloying on the modulation of intermediates and the catalytic mechanism during CO_2_RR, we performed in situ attenuated total reflection surface‐enhanced infrared (ATR‐SEIR) spectroscopy measurements in the identical acidic electrolyte conditions (0.05 m H_2_SO_4_ and 0.5 m K_2_SO_4_, pH 1). Infrared (IR) spectra were collected for four catalysts—Cu, Cu_3_Sn, Cu_6_Sn_5_, and Sn—over a potential range of −0.3–1.2 V. As shown in **Figure** **5**a–d, IR absorption peaks near 1390 cm^−1^ were detected on the surface of all four catalysts. These peaks are attributed to the symmetric stretching vibration of the CO_2_RR intermediate species ^*^OCHO (νC═O).^[^ ^4^, ^42^, ^43^, ^49^ ^]^ Furthermore, as the potential varied, a distinct Stark shift in Figure 5e confirms the presence of intermediate species adsorbed on the catalyst surface.^[^ ^50^, ^51^ ^]^ The IR peaks of the ^*^OCHO intermediate on Cu_6_Sn_5_ and Cu_3_Sn catalysts display a distinct redshift compared to those on Cu and Sn catalysts over the potential range of −0.3–1.2 V. This redshift suggests a weaker C─O bond, likely due to enhanced interaction or adsorption of the ^*^OCHO intermediate.^[^ ^50^ ^]^ Notably, the Cu_6_Sn_5_ catalyst exhibits the most pronounced shift, with the ^*^OCHO absorption band shifting by 5 cm^−1^ relative to Cu_3_Sn at −0.6 V. For quantitative analysis of the in situ ATR‐SEIR spectra, the area under the ^*^OCHO peak was normalized against the maximum observed O─H stretching bands of the adsorbed water (Figure 5f; Figure S9, Supporting Information). The increase in ^*^OCHO absorption bands on the surface of the Cu_6_Sn_5_ catalyst is significantly faster than that of the other catalysts. Quantitative analysis further reveals that the peak area of ^*^OCHO absorption bands is markedly enhanced on the CuSn alloy surfaces compared to the Cu and Sn catalysts. Specifically, at −1 V, the peak area of the ^*^OCHO band for Cu_6_Sn_5_ was 1.85, 4.18, and 1.43 times higher than that for Cu, Sn, and Cu_3_Sn, respectively (Figure 5f). Furthermore, Figure 5c shows that no significant ^*^CO signal was detected on the surface of Cu_6_Sn_5_, indicating that the competing pathways for CO and C─H product formation were effectively suppressed. These findings suggest that the stronger adsorption capacity of Cu_6_Sn_5_ results in higher surface coverage of ^*^OCHO, thereby favoring the reduction of CO_2_ to formic acid.

### 2.4 Bias‐Free PEC‐CO_2_RR Performance

**Figure 6:**
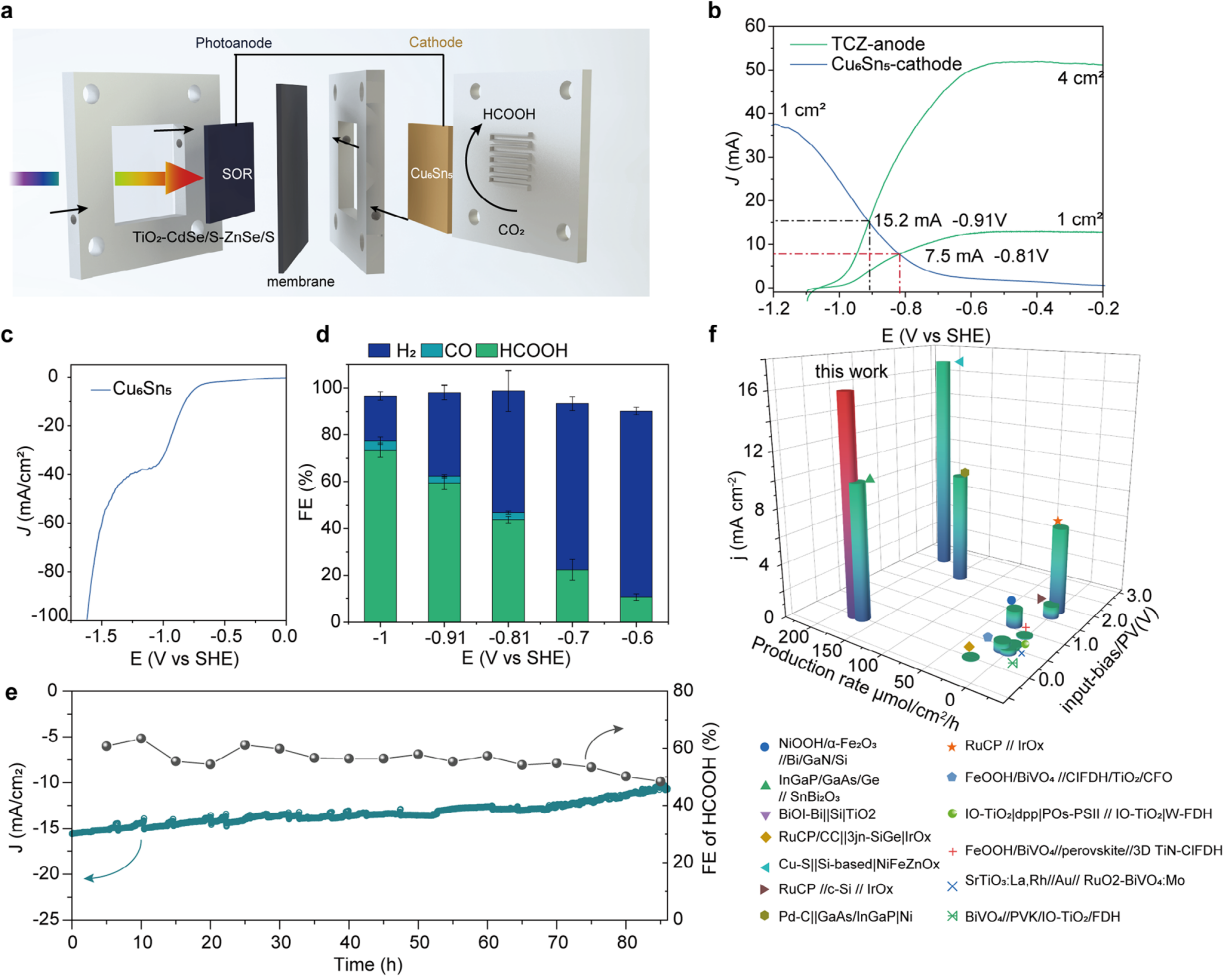
PEC‐CO_2_RR system. a) Schematic diagram of the PEC‐CO_2_RR flow cell setup for the unbiased two‐electrode system. b) Current–voltage characteristics of the TCZ photoanode and Cu_6_Sn_5_ cathode. c) LSV curves for electrocatalysis and d) product distribution of the Cu_6_Sn_5_ cathode in 0.05 m H_2_SO_4_ + 0.5 m K_2_SO_4_. Error bars represent standard deviations from three replicate experiments. e) Stability test results of the TCZ//Cu_6_Sn_5_ system and the two‐electrode PEC‐CO_2_RR. f) Performance comparison with other PEC‐CO_2_RR systems for formic acid production.

To construct a bias‐free PEC‐CO_2_RR system, we integrate the TCZ photoanode and Cu_6_Sn_5_ electrocathode into a pH‐decoupled PEC flow cell utilizing the chemical potential difference between the anode and cathode to assist the solar‐driven conversion of CO_2_ to HCOOH (**Figure** **6**a). Due to the pH difference between the anolyte and catholyte, we normalize the working potential of the PEC system to the standard hydrogen electrode (SHE) rather than the reversible hydrogen electrode (RHE) to avoid pH interference, as shown in Equation (1).

(1) $$E_{\mathrm{SHE}}=E_{\mathrm{appl}\mathrm{ied}}+E_{\mathrm{Ag}/\mathrm{AgCl}}=E_{\mathrm{RHE}}-0.0592\times \mathrm{pH}$$

Under AM 1.5G solar illumination, the current–voltage curves of the TCZ photoanode (1 cm^2^) in alkaline 0.5 m Na_2_SO_3_ + Na_2_S electrolyte and the Cu_6_Sn_5_ cathode (1 cm^2^) in 0.05 m H_2_SO_4_ acidic electrolyte show that the integrated system operates at a potential of −0.81 V versus SHE with a current of 7.5 mA cm^−2^ (based on the area of the cathode catalyst) (Figure 6b). As seen in Figure 6c,d, the selectivity and reaction rate for formic acid at this condition is suboptimal (with a formic acid FE of 43%) due to the mismatch between the operating potential and optimal potential of the Cu_6_Sn_5_ catalyst. To solve the problem of potential mismatch, we increased the TCZ photoanode area to 4 cm^2^ and kept the cathode area (1 cm^2^) unchanged. The working potential of the PEC system has been raised to −0.91 V versus SHE, increasing the formic acid FE to 61% and raising the operating current to 15.2 mA cm^−2^ (Figure 6b). During 85 h of testing under simulated AM 1.5G solar illumination without external bias, the PEC system maintained an average current of 14 mA cm^−2^, with a cathodic formic acid FE of 60% (Figure 6e), presenting excellent stability. Moreover, the system achieves a formic acid production rate of 179 µmol cm^−2^ h^−1^. Notably, the performance of this device far exceeds that of state‐of‐the‐art solar‐driven formate production systems based on traditional OER‐CO_2_RR photoelectrolysis (Figure 6f; Table S2, Supporting Information).

## 3 Conclusion

In this study, we present a systematical design principle for an unbiased PEC‐CO_2_RR system to produce liquid fuel. In terms of material, we expand the absorption spectrum of the photoanodes and promote the separation of photogenerated carriers by constructing gradient energy bands and nanoarchitecture. By adjusting the adsorption capability for the *OCHO intermediates, the optimized catalyst demonstrates an FE of 92% and stability for up to 100 h. Furthermore, from the electrode and system configuration aspects, we develop a pH decoupled PEC‐CO_2_RR to fully utilize chemical potential to counteract limited power output, where the sluggish OER is replaced by the SOR, thereby promoting the reaction kinetics and reducing the reaction potential. Finally, the PEC‐CO_2_RR system delivers a formic acid yield of up to 172.9 µmolh^−1^cm^−2^ over an 85‐h long‐term test, surpassing the performance of conventional solar‐driven systems. These findings offer a cost‐effective strategy for solar‐driven PEC‐CO_2_RR systems.

## Conflict of Interest

The authors declare no conflict of interest.

## Supporting information



Supporting Information

## Data Availability

All data supporting the findings of this study are available within the article and its Supplementary Information, and can also be obtained from the corresponding author upon reasonable request.
