# Supplementary material for: Architecting a Bias‐Free Photoelectrochemical CO2 Reduction System for Sustainable Formic Acid
Source: Adv Sci (Weinh). 2025 Apr 4;12(21):2415774. doi: 10.1002/advs.202415774 (PMC12140357; doi:10.1002/advs.202415774)
Supplement: Supplementary file 1 — Supporting Information [file ADVS-12-2415774-s001.docx]

**Supporting Information**

**Architecting a Bias-Free Photoelectrochemical CO_2_ Reduction System for Sustainable Formic Acid**

*Yinchao Yao^1^, Zilong Wu^2^, Zhiwei Zhao^1^, Zhiyi Sun^1^, Tiesong Li^1^, Zebiao Li^3^, Xinxin Lu^3^,and Zhuo Chen^1^**

^1^Energy & Catalysis Center, School of Materials Science and Engineering, Beijing Institute of Technology, Beijing 100081, PR China.

^2^China Academy of Information and Communications Technology, No. 40, Huayuan Road, Haidian District, Beijing 100190, PR China.

^3^PetroChina Shenzhen New Energy Research Institute Co., Ltd., Shen Zhen 518052, PR China.

**1.Experimental Section**

**1.1 Materials**

All the chemical reagents, which are analytically pure, were used as purchased without further purification. Reagents include Cd(NO_3_)_2_, Zn(NO_3_)_2_, Na_2_SeSO_3_, Se powder, SnSO_4_, CuCl_2_, SnCl_2_, Na_4_P_2_O_7_, CH_3_COONH_4_, K_2_SO_4_ and NH_4_F were purchased from Shanghai Aladdin Biochemical Technology Co., Ltd. H_2_SO_4_, HCl, Acetone and ethanol purchased from Beijing Tongguang Fine Chemicals Company.

**1.2 Fabrication of TiO_2_ nanotube arrays**

TiO_2_ nanotube arrays were prepared using a two-step anodization process in an electrolyte consisting of ethylene glycol with 0.3 wt.% NH_4_F and 2 wt.% H_2_O. Titanium foils (99.4% purity, 0.1 mm thickness) and titanium mesh (20 and 40 mesh) were used. The process began with ultrasonic cleaning of the titanium foils in chloroform, acetone, and ethanol for 10 minutes each, followed by drying. The first anodization was carried out at 60 V for 1 hour, using the titanium foil as the anode and a graphite plate as the cathode. After a uniform oxide layer formed, the samples were cleaned in a 1:1 ethanol-deionized water solution to remove the oxide. The second anodization, also at 60 V but for 2 hours, was conducted while maintaining the electrolyte at 5 ℃ to ensure uniform growth of titanium dioxide nanotubes. Post-anodization, the samples were immersed sequentially in ethylene glycol, a 1:1 mixture of ethylene glycol and acetone, and pure acetone, each for 30 minutes, followed by rinsing and drying. Finally, the samples were annealed in a muffle furnace at 450°C for 1 hour, with a 1-hour ramp-up time, and then cooled naturally.

**1.3 Fabrication of TCZ and TC photoanodes**

The TCZ photoanodes were fabricated via successive ionic layer adsorption and reaction (SILAR), followed by annealing in a tubular furnace under an argon and 7% H_2_ atmosphere. The TiO_2_ nanotube arrays were prepared through a two-step anodization process; see Supplementary Methods for details. The TiO_2_ nanotube arrays were first immersed in a 0.1 M cationic solution (Cd^2+^ or Zn^2+^) for cation adsorption, followed by rinsing with methanol. Subsequently, the samples were immersed in a 0.1 M anionic solution (S^2-^ or Se^2-^) for 1 minute to adsorb the anions. The Cd^2+^ source was a 0.1 M Cd(NO_3_)_2_ methanol solution, the Zn^2+^ source was a 0.1 M Zn(NO_3_)_2_ methanol solution, the S^2-^ source was a 0.1 M Na_2_S solution in a methanol-water mixture (7:3, v/v), while the Se^2^⁻ source, Na_2_SeSO_3_, was prepared by dissolving 0.1 M selenium powder and 0.15 M Na_2_SO_3_ in a water-methanol mixture. After each adsorption step, the samples were rinsed with methanol to remove excess ions. Finally, the electrodes were annealed under H_2_ (5%) /Ar atmosphere at a heating rate of 5 ℃ min^-1^ up to 400°C, held at this temperature for 1 hour, and then cooled to room temperature. The control sample (TC electrode) was prepared without ZnSe/S loading.

**1.4 Synthesis of CuSn alloy catalysts**

The CuSn alloy catalysts (Cu, Cu_3_Sn, Cu_6_Sn_5_, and Sn) were synthesized via constant current electrodeposition using a three-electrode system on a Chi660E electrochemical workstation. A 1×1 cm^2^ copper foam served as the working electrode, a 4×4 cm^2^ copper foil as the counter electrode, and Ag/AgCl as the reference electrode. Before deposition, both electrodes were cleaned sequentially with acetone, ethanol, 0.1 M HCl, and deionized water. Cu_6_Sn_5_ was prepared using Electrolyte A^[1]^ (0.015 M CuSO_4_, 0.015 M SnSO_4_, 0.15 M K_2_SO_4_, 0.03 M H_2_SO_4_) at –1 A cm⁻^2^ for 5 minutes, with stirring at 800 rpm. Cu_3_Sn was deposited from Electrolyte B^[2]^ (0.01 M CuCl_2_, 0.01 M SnCl_2_, 0.01 M Na_4_P_2_O_7_, 0.01 M CH_3_COONH_4_, 0.5 M HCl) at –2 mA cm⁻^2^ for 60 minutes. The Cu catalyst was obtained from an electrolyte containing 0.03 M CuSO_4_, 0.15 M K_2_SO_4_, and 0.03 M H_2_SO_4_, following the same deposition conditions as for Cu_6_Sn_5_. Sn catalysts were deposited using a Sn foil as the counter electrode, carbon paper as the working electrode, and Ag/AgCl as the reference electrode, with electrodeposition carried out at –1 A cm⁻^2^ for 5 minutes.

**1.5 Materials Characterizations**

A Hitachi SU4800 scanning electron microscope (SEM) was employed to capture SEM images of the samples at an accelerating voltage of 5 kV. Subsequently, to obtain more detailed insights into the microstructure, a Tecnai F20 transmission electron microscope (TEM) was utilized. High-resolution TEM (HRTEM) analysis, TEM-energy dispersive X-ray spectroscopy (TEM-EDS), and selected area electron diffraction (SAED) were conducted at an accelerating voltage of 200 kV. The crystal structure of the samples was examined using a Rigaku Smartlab X-ray powder diffractometer (XRD), with XRD patterns collected over a 2θ range of 20° to 80° at a scanning rate of 5°/min. The surface chemical composition was analyzed via XPS using a PHI QUANTERA-II SXM instrument, and the binding energy data were calibrated against the C 1s peak, set at a reference value of 284.6 eV.

**1.6 Electrochemical and photoelectrochemical measurements**

Electrochemical measurements, including cyclic voltammetry (CV), linear sweep voltammetry (LSV), electrochemical impedance spectroscopy (EIS), and chronopotentiometry (CP), were conducted using a Chenhua electrochemical workstation (model Chi 660e). The photoanode samples were used as the working electrode, with the electrolyte consisting of a mixed solution of 0.25 M Na_2_S and 0.35 M Na_2_SO_3_. A saturated calomel electrode (SCE) served as the reference electrode, and a platinum electrode was employed as the counter electrode. The contact area between all samples and the electrolyte was maintained at 1 cm^2^.EIS and open-circuit voltage (OCV) measurements were performed using the same electrochemical workstation, with EIS data analyzed using the Z-simp win 3.1 software. EIS tests were performed at an open circuit potential over frequencies ranging from 1 Hz to 100000 Hz. Incident light-to-current conversion efficiency (IPCE) spectra were measured using a QE/IPCE measurement system (Crowntech QTest Station 1000AD), equipped with a tungsten-halogen lamp (CT-TH-150), a standard silicon diode, and a monochromator (Crowntech QEM24-S 1/4 m).

The electrocatalytic CO_2_ reduction reaction tests were performed in a three-electrode flow cell, where a Nafion proton exchange membrane (Nafion 117) was used to separate the cathode and anode chambers, facilitating ion conduction. A gas diffusion electrode (GDE) loaded with a CuSn alloy catalyst (loading amount: 1 mg cm^-2^) served as the working electrode, with an active area of 1 cm^2^. The reference electrode was an Ag/AgCl electrode, and the counter electrode was a Ti mesh coated with IrO_2_, also with an effective area of 1 cm^2^. The electrolyte consisted of 0.05 M H_2_SO_4_ and 0.5 M K_2_SO_4_. The CO_2_ flow rate was maintained at 30 sccm, regulated by a mass flow controller (Sevenstar D07). The electrochemical surface area (ECSA) of the CuSn alloy was evaluated by measuring CV curves at scan rates of 5, 10, 20, 40, and 80 mV/s in 0.05 M H_2_SO_4_ electrolyte. The measurements were conducted within a non-Faradaic potential window of -0.018 to -0.058 V vs Ag/AgCl. The ECSA was determined by comparing the double layer capacitance (C_dl_), which was extracted from the slope of the current density versus the scan rate plot, as an indicator of the material’s electrochemical active surface area.

The photoelectrochemical (PEC) CO_2_ reduction tests were conducted in a dual-chamber flow cell equipped with either a Nafion proton exchange membrane (Nafion 117) or a bipolar membrane as the separator. The TCZ was used as a photoanode, while the cathode was a Cu_6_Sn_5_-based gas diffusion electrode (GDE) with a catalyst loading of 1 mg cm^-2^. The electrolyte in the anodic chamber consisted of 0.25 M Na_2_SO_3_ and 0.5 M Na_2_S, while the cathodic chamber contained 0.05 M H_2_SO_4_ and 0.5 M K_2_SO_4_. During the tests, simulated sunlight from a xenon lamp(AM 1.5 G,100 mW cm^-2^ ) illuminated the photoanode, and a closed circuit was formed between the anode and cathode via a connecting wire, enabling CO_2_RR on the cathode.

All potentials mentioned in this work (unless otherwise specified) were converted to the reversible hydrogen electrode (RHE) scale using the Nernst equation, as follows:

E_RHE_=E_Ag/AgCl_ + 0.059×pH +0.197 =E_SCE_ + 0.059×pH +0.244 (S.1)

**1.7 CO_2_RR Product analysis**

The gaseous products of the electrochemical and photoelectrochemical reduction were analyzed using gas chromatography (GC) with a Hui Fen 9890E instrument. The GC system was equipped with a thermal conductivity detector (TCD) and a hydrogen flame ionization detector (FID) for online detection of gas-phase products. Liquid-phase products were characterized by nuclear magnetic resonance (NMR) spectroscopy. The NMR samples were prepared by mixing 450 μL of the electrolyte, 50 μL of D_2_O, and 50 μL of 20 mM DMSO as an internal standard. Proton NMR (^1H-NMR) spectra were acquired using the H_2_O suppress mode to quantify the liquid-phase products.

The FE of the products was calculated using the following equation:

$\text{FE}_{\text{gas}}\text{=}\frac{\text{Q}_{\text{gas}}}{\text{Q}_{\text{total}}}\text{=}\frac{\text{n}_{\text{gas}}\text{NF}}{\text{Q}_{\text{total}}}\text{=}\frac{\text{(}\frac{\text{v}}{\text{60}\text{ }\text{s/min}}\text{)×(}\frac{\text{y}}{\text{22400}\text{cm}^{\text{3}}\text{/mol}}\text{)NF}}{\text{j}}\text{×100\%}$ (S.2)

$\text{FE}_{\text{liquid}}\text{=}\frac{\text{Q}_{\text{liquid}}}{\text{Q}_{\text{total}}}\text{=}\frac{\text{n}_{\text{liquid}}\text{NF}}{\text{Q}_{\text{total}}}\text{×100\%}$ (S.3)

*v* is the gas flow rate measured by the flow meter, *y* is the volume fraction of the gaseous product, N represents the number of electrons transferred for each product, F denotes Faraday's constant (96500 C mol^-1^), j denotes the current, t corresponds to the running time, and N_liquid_ (in moles) stands for the amount of the liquid product determined by 1H- NMR

**1.8 Formula for ABPE efficiency**

$\text{η=I×(1.23}\text{-}\text{V)/}\text{J}_{\text{light}}$ （S.4）

where I is the photocurrent density at the corresponding test potential, V is the applied bias voltage (V vs RHE), and _Jlight_ is the light intensity at 100 mW cm^-2^.

**1.9 The formula for calculating IPCE**

$\mathrm{IPCE}\left( \% \right)=[1240\times I\left( {mA cm}^{-2} \right)]/[\lambda(nm){\times J}_{\mathrm{light}}({W cm}^{-2})]$ （S.5）

I denote the photocurrent density measured at a specific wavelength λ while J_light_ represents the irradiance intensity at the same wavelength.

**1.10 Mott-Schottky measurements**

To further investigate the electrochemical mechanism of the photoanode samples, Mott-Schottky analysis was conducted in the dark at a frequency of 1000 Hz. This analysis provides insights into the semiconductor type, flat-band potential, and carrier concentration of the photoanode materials. The specific calculation process is presented in Equation :

$\frac{\text{1}}{\text{c}^{\text{2}}}\text{=(E-}\text{E}_{\text{FB}}\text{-}\frac{\text{kT}}{\text{e}}\text{)/}\text{N}_{\text{d}}\text{e}\text{ε}_{\text{0}}\text{ε}$ （S.6）

For n-type semiconductors, the carrier concentration (N_d_) can be derived from the Mott-Schottky plot using Equation (S4):

$\text{N}_{\text{d}}\text{=2(}\frac{\text{dE}}{\text{d}\left（ \frac{\text{1}}{\text{c}^{\text{2}}} \right）}\text{)/(e}\text{ε}_{\text{0}}\text{ε}\text{A}^{\text{2}}\text{)}$ （S.7）

Where N_d_ is the carrier concentration, c is the semiconductor space charge capacitance, E is the applied bias voltage, e is the electron charge, A is the contact area between the sample and the liquid, ε_0,_ and ε_r_ are the vacuum permittivity and the material relative permittivity, respectively, E_pe_ is the flat band potential, K is Boltzmann's constant, and T is the thermodynamic temperature.

**1.11 *In situ* surface-enhanced infra-red absorption spectroscopy (SEIRAS) measurement**

SEIRAS measurements were conducted in a two-compartment spectro-electrochemical cell, with a Nafion membrane separating the compartments. An Ag/AgCl electrode was employed as the reference electrode, while the working electrode consisted of a metal film deposited on a Si ATR prism. A platinum wire was used as the counter electrode. Spectra were acquired by co-adding 128 scans at a spectral resolution of 4 cm^−1^. All measurements were performed using a Bruker INVENIO S FTIR spectrometer equipped with a liquid-nitrogen-cooled mercury cadmium telluride (MCT) detector.

Au film underlayers were chemically deposited onto Si ATR crystals following established protocols. The prism was first polished with a 0.05 μm Al_2_O_3_ slurry and subsequently cleaned via ultrasonication in acetone and deionized water. To generate a hydrogen-terminated surface, the reflecting plane of the prism was immersed in a 40% NH_4_F solution for 5 minutes. It was then transferred into a solution containing 2% HF and a gold plating mixture comprising 5.75 mM NaAuCl_4_·2H_2_O, 0.025 M NH_4_Cl, 0.025 M Na_2_S_2_O_3_·5H_2_O, 0.075 M Na_2_SO_3_, and 0.026 M NaOH at 60°C for 10 minutes^[3]^. The Au-coated Si prism was rinsed with deionized water and dried under ambient conditions. The Au film was electrochemically cleaned and activated using cyclic voltammetry (CV)^[4]^ in 0.1 M H_2_SO_4_. Four CV cycles were performed by sweeping the potential from 0.1 to 1.5 V versus RHE at a scan rate of 50 mV s^-1^. CuSn alloy electrodes were fabricated by depositing CuSn alloy ink onto the activated Au film. The ink was prepared by dispersing 100 mg of commercial CuSn alloy powder in 2.5 mL of isopropanol, followed by the addition of 40 μL of 5% Nafion solution. The mixture was sonicated for 30 minutes to ensure uniform dispersion. Finally, 75 μL of the ink was evenly applied to a 1 cm^2^ area of the Au film and left to dry at room temperature overnight.

**1.12 Statistical Analysis**

The data presented were the raw data unless otherwise stated below or in the text. Normalization was performed on the *OCHO peak area (normalized to the largest ν(O−H) band of *H_2_O) in the ATR-SEIRS spectrum, as shown in Figure 5f. Averaging and statistical analysis of the estimated compositions are provided in the Supporting Information or within the text where appropriate.

**2.Supporting figures and tables**


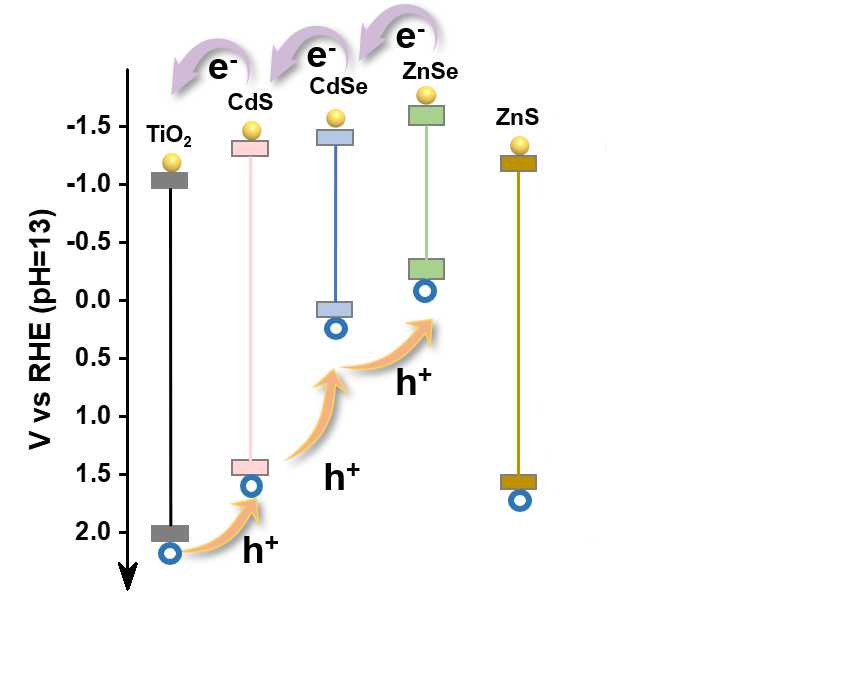


**Figure S1.** Schematic diagram of the energy band structure of the TiO_2_-CdS/Se-ZnSe/S photoanode.

(1) The TiO_2_-loaded CdS/Se-ZnSe/S quantum dots enables wider absorption for solar energy; (2) The gradient energy band structure enhances the transport and separation efficiency of photogenerated carriers, as shown in **Figure S1**, significantly increasing the current density of the photoanode. The TiO_2_/CdS/CdSe/ZnSe heterostructure exhibits a well-ordered gradient energy level arrangement, where the photogenerated electrons from CdS/CdSe/ZnSe tend to follow the gradient energy levels. Specifically, the electrons move from the ZnSe conduction band (which has a higher barrier energy level) to CdSe, then to CdS, and ultimately to TiO_2_, where they are transported through the external circuit to the cathode for the reduction reaction. The TiO_2_/CdS/CdSe/ZnSe heterostructure provides energy barriers that inhibit electron back-transfer; (3) The ZnS layer, with its more positive valence band position, is less prone to oxidation and dissolution. It functions as a passivation layer, thus improving the stability of quantum dots. Additionally, holes can reach the interface for oxidation reactions via the 'tunneling effect,' overcoming the high barrier of the thin passivation layer.


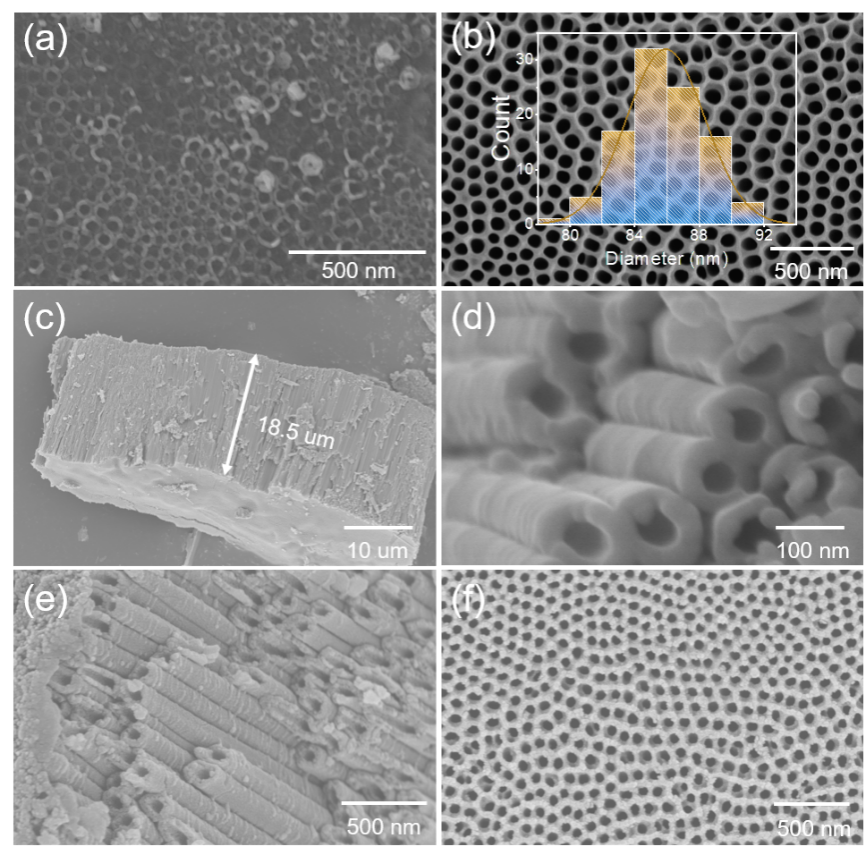


**Figure S2.** (a) SEM image of the porous structure formed after removing TiO_2_ nanotubes, (b) SEM image of the front side of TiO_2_ nanotube array, (c) SEM image of the side view of TiO_2_ nanotube array, (d) and (e) high-resolution SEM images of TiO_2_ nanotubes, (f) SEM image of the sensitized photoanode sample


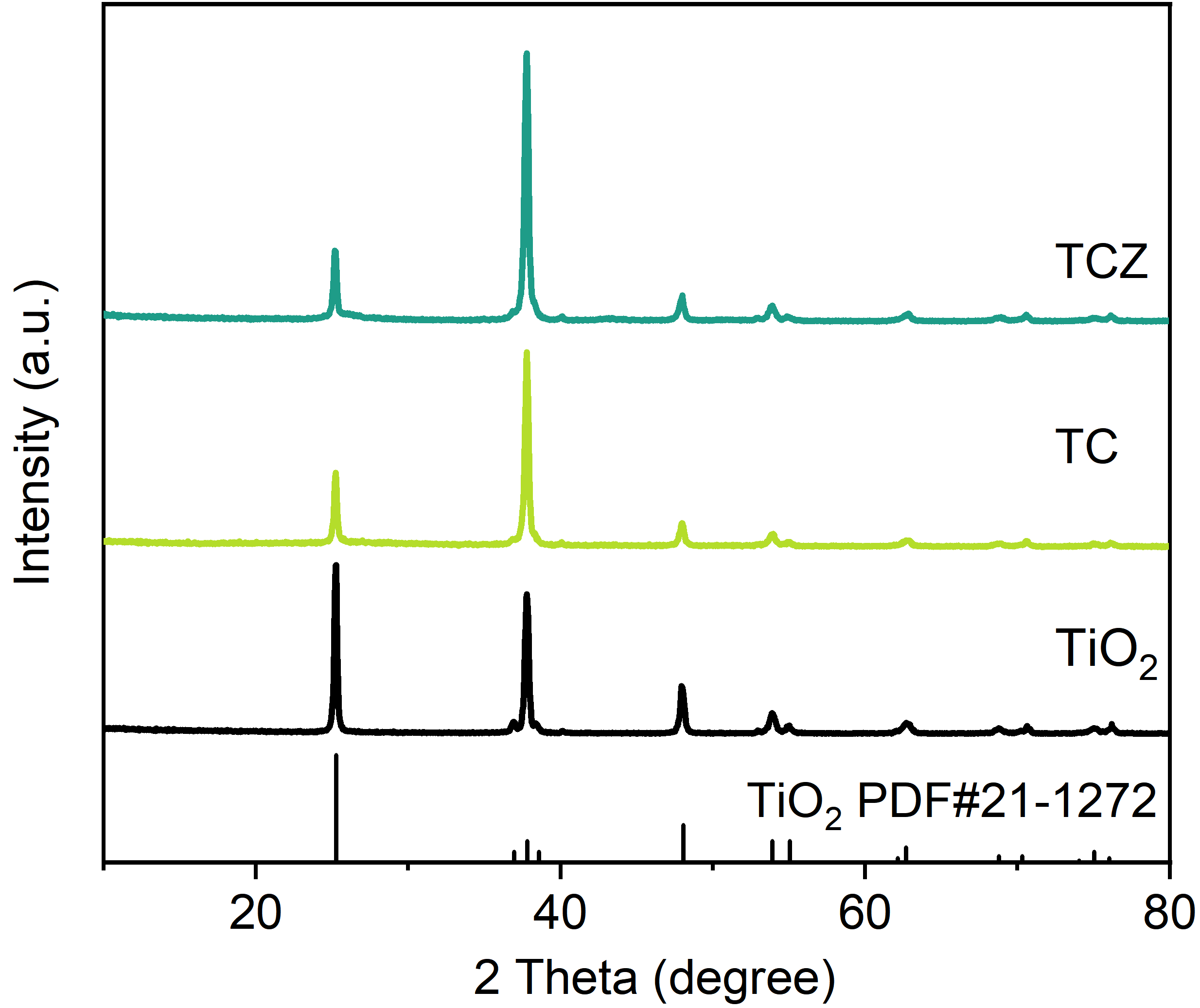


**Figure S3.** XRD patterns of different photoanode samples


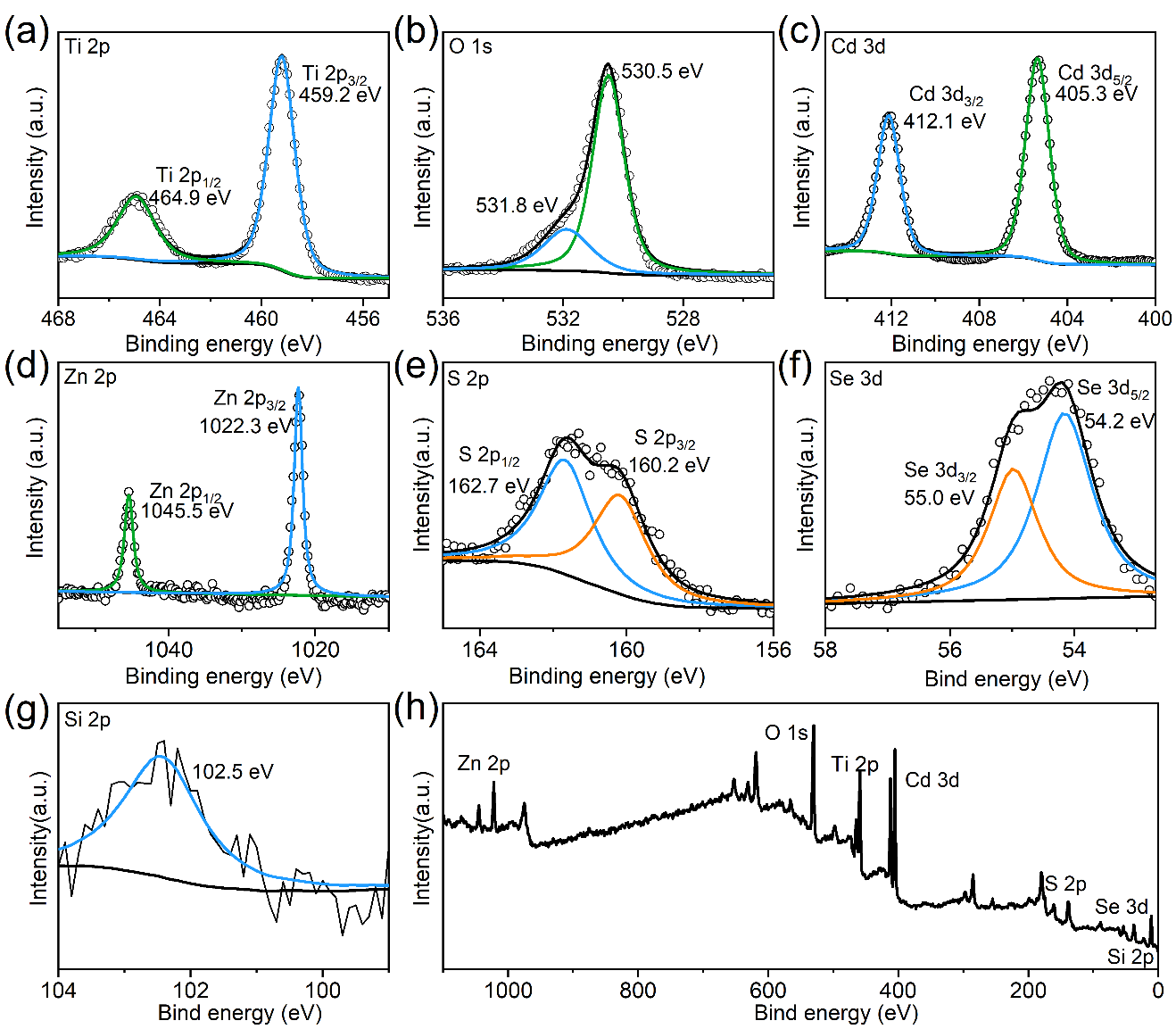


**Figure S4.** XPS spectra of TCZ photoanode sample: (a) Ti 2*p* XPS spectrum, (b) O 2*p* XPS spectrum, (c) Cd 3*d* XPS spectrum, (d) Zn 2*p* XPS spectrum, (e) S 2*p* XPS spectrum, (f) Se 3*d* XPS spectrum


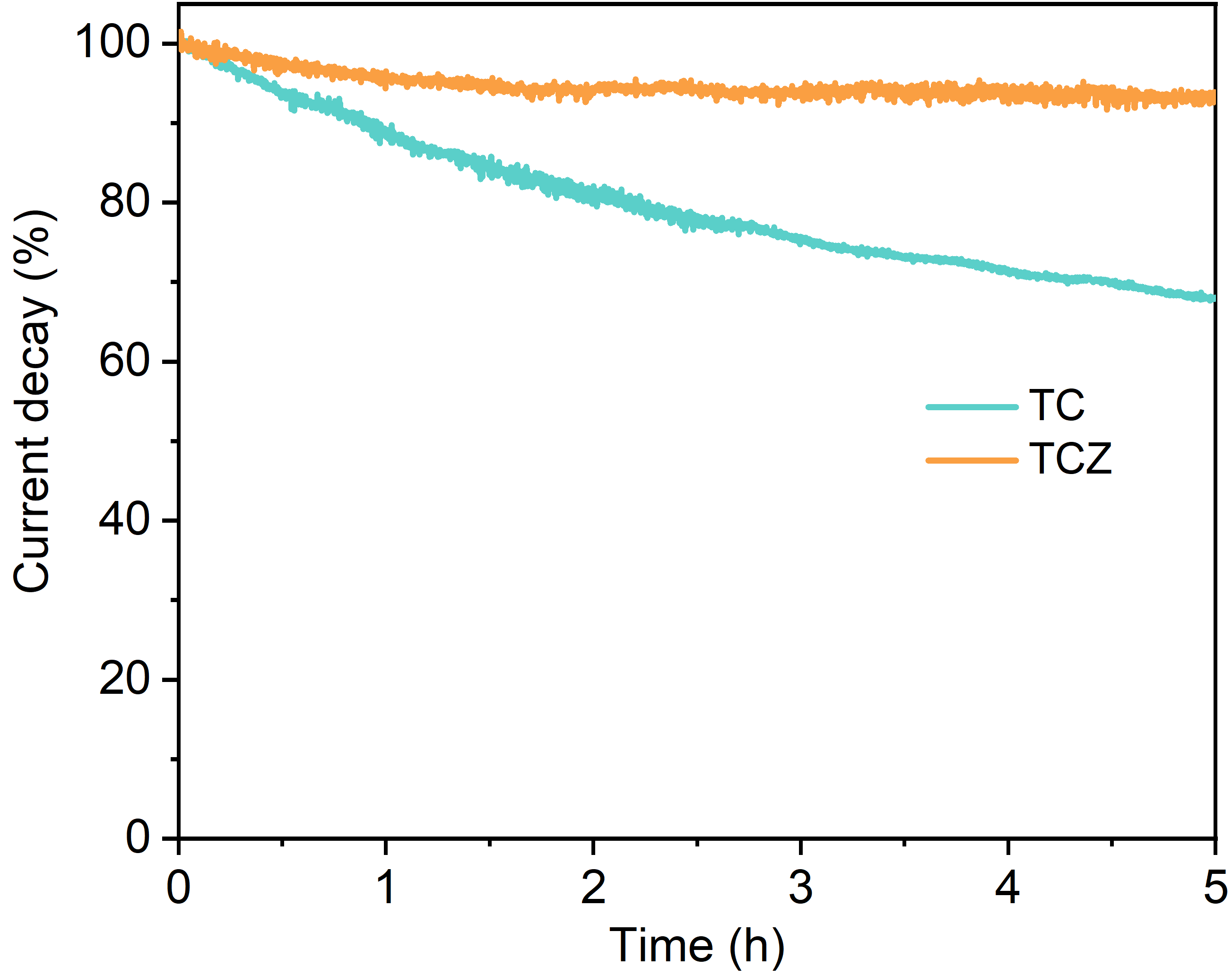


**Figure S5.** Stability test of TC and TCZ photoanodes.

**Table S1**. Photoelectrochemical properties for different photoanode samples

| Catalysis | EIS | | | | | photoelectron lifetimes | carrier concentration |
| --- | --- | --- | --- | --- | --- | --- | --- |
|  | R_s_ | C_bulk_*(10^-8^) | R_trapping_ | C_trap_*(10^-4^) | R_ct,trap_ |  |  |
| TC | 3.36 | 3.05 | 24.84 | 2.49 | 5847 | 8.6s | 6.48*10^19^ cm^-3^ |
| TCZ | 3.54 | 2.98 | 25.10 | 2.54 | 3238 | 8.9s | 9.43*10^19^ cm^-3^ |


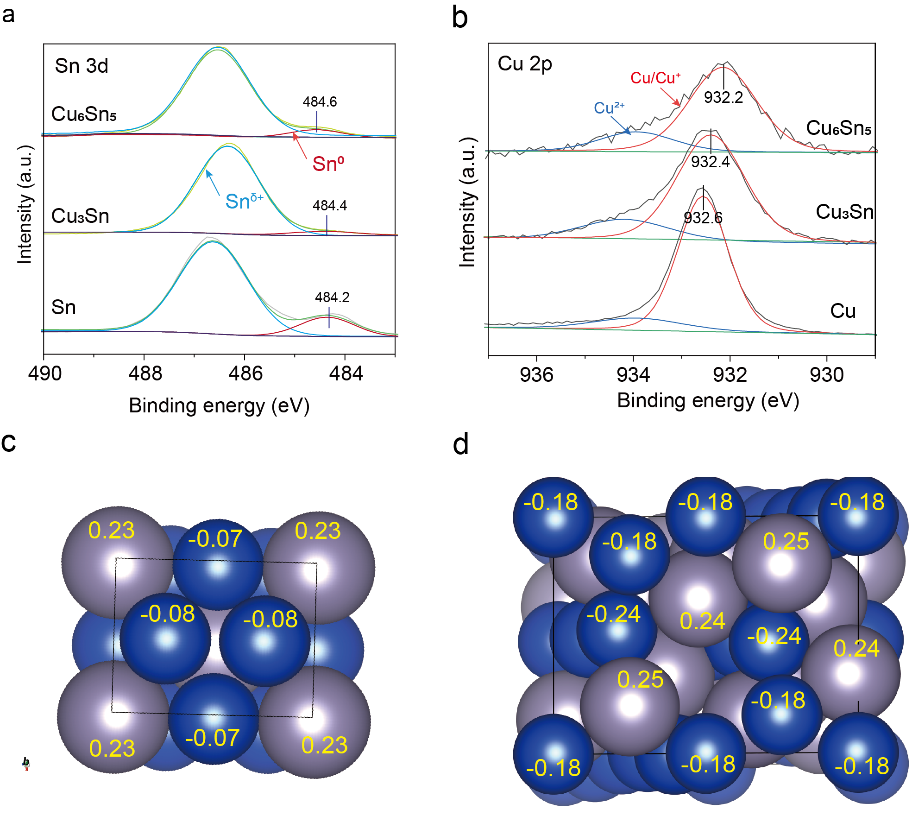


**Figure S6.** The a) Sn 3d and b) Cu 2p Spectra of Cu, Sn and CuSn alloys. The optimized unit cell and corresponding Bader valence for c) Cu_3_Sn, d) Cu_6_Sn_5_. Blue and grey spheres indicate Cu and Sn atoms, respectively

All the spin-polarized DFT calculations were carried out using Vienna Ab-initio Simulation Package (VASP)^[5, 6]^ code with the Perdew-Burke-Ernzerhof (PBE) functional^[7, 8]^. The project-augmented wave (PAW)^[9]^ method with plane wave basis sets was applied to treat the core-valence electron interaction. The optimized Unit Cell of Cu_3_Sn and Cu_6_Sn_5_ contain 8 (6 Cu atoms and 2 Sn atoms) and 44 (24 Cu atoms and 20 Sn atoms) atoms, respectively. During the optimization of the unit cell, the cut-off energy was 520 eV, and the atoms were relaxed until the forces on each atom were less than 0.05 eV Å^-1^. The k-points for Cu_3_Sn and Cu_6_Sn_5_ optimization was set as 5*5*4 and 2*3*2. The lattice parameter of optimized unit cell (Cu_3_Sn: a=4.32 Å, b = 4.79 Å, c = 5.61 Å, α = 90.00°, β = 98.67°, γ = 90.00°; Cu_6_Sn_5_: a=11.13 Å, b = 7.52 Å, c = 9.81 Å, α = 90.00°, β = 98.67°, γ = 90.00°)


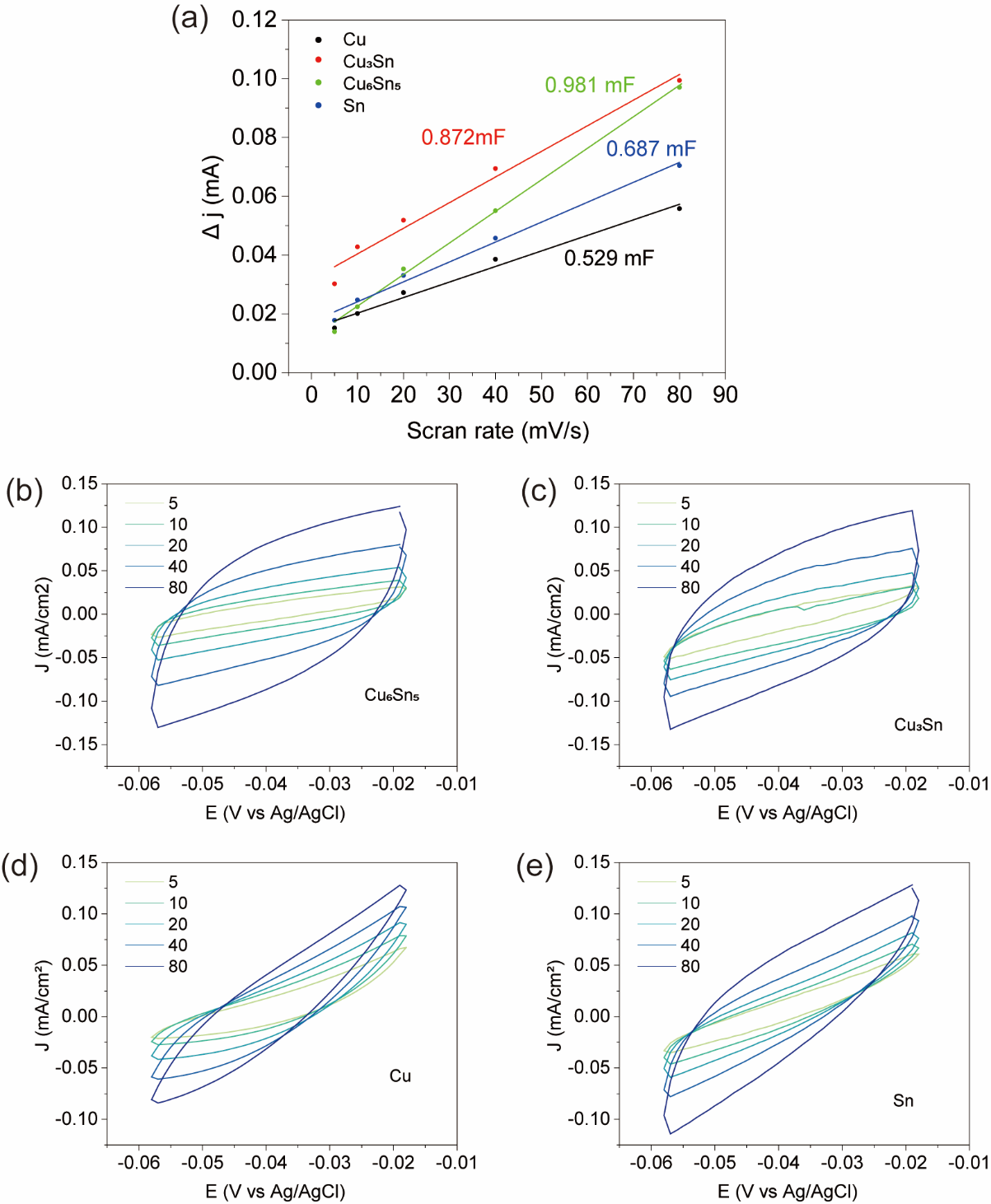


**Figure S7.** (a),Double layer capacitance of the Cu, Cu_3_Sn, Cu_6_Sn_5_, Sn electrode surfaces, (b-e), CVs with various scan rates.

**Table S2**. ICP-MS results for the electrolyte after a 120-hour stability test.

| Sample | Cu concentration (ug/L) | Sn concentration (ug/L) |
| --- | --- | --- |
| 100 mL electrolyte | 12.73±0.39 | 2.57±0.14 |


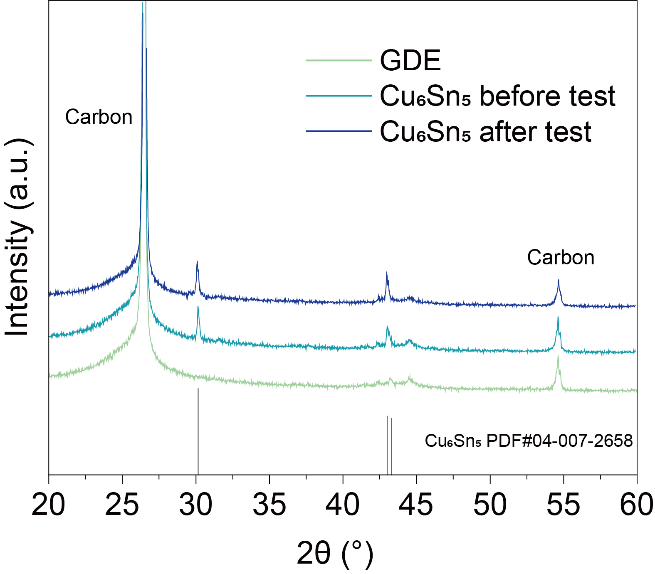


**Figure S8**. XRD pattern of Cu_6_Sn_5_ catalyst before and after 120-hour stability test.


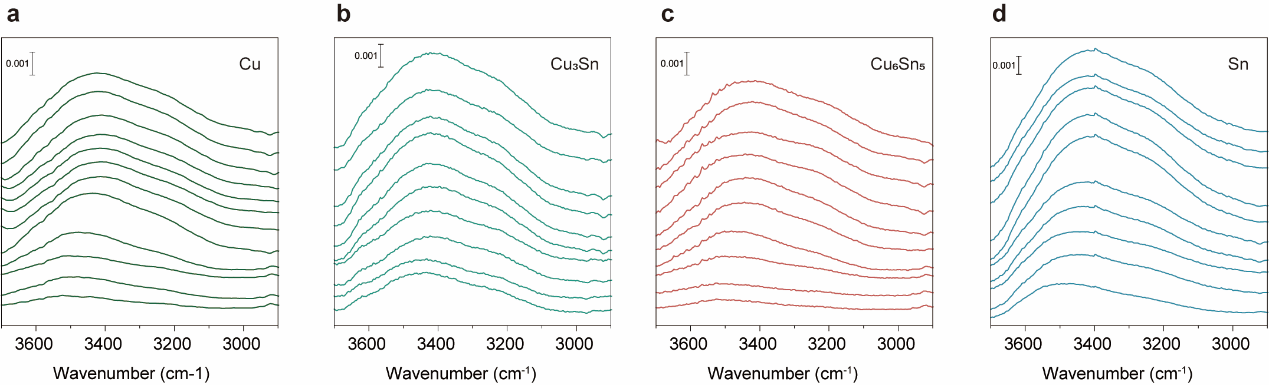


**Figure S9.** in-situ SEIRS of (a), Cu, (b)Cu_3_Sn, (c)Cu_6_Sn_5_, (d)Sn catalysts in the wavenumber of 3000-3700cm^-1^.


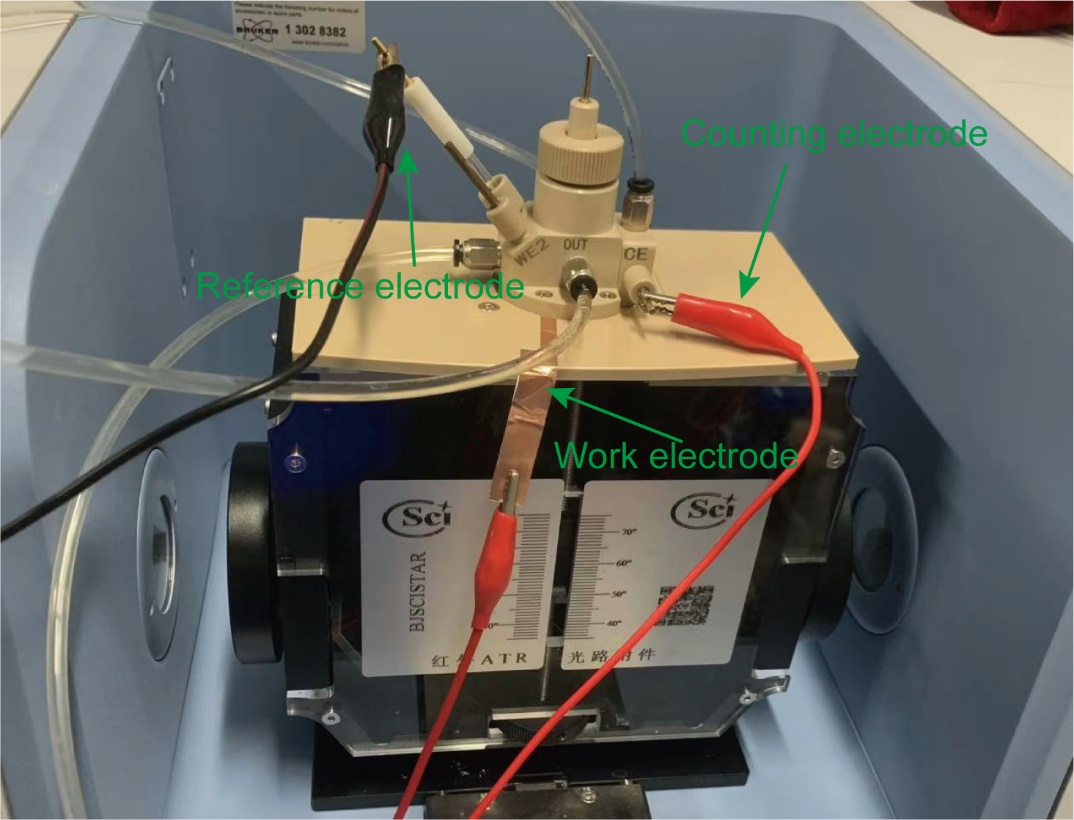


**Figure S10.** A photograph of *in situ* SEIRAS setup in this work.


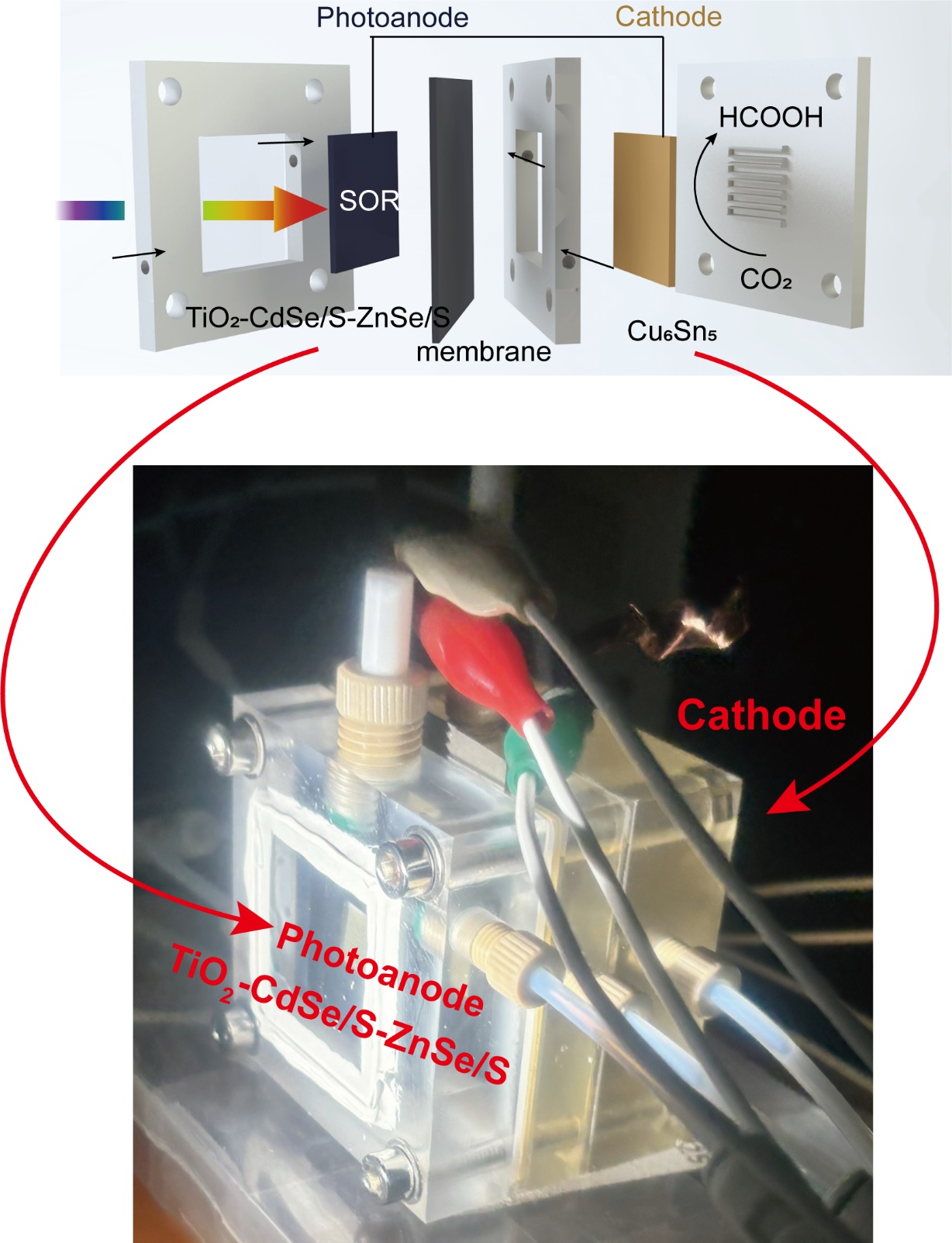


**Figure S11.** A photograph of the two-electrode TCZ//Cu_6_Sn_5_ and two-electrode photoelectrochemical CO_2_ reduction reaction setup utilized in this study.

**Table S3.** Performance comparison of PEC-CO_2_RR systems for formic acid preparation.

| catalysts ( cathode// anode) | Production rate μmol/cm^2^/h | current density mA/cm^2^ | FE | External bias（V） | Stability(h) | electrolyte | ref |
| --- | --- | --- | --- | --- | --- | --- | --- |
| Cu_6_Sn_5_//TCZ | 172.9 | 15.2 | 61 | bias-free | 85 | 0.05 M H_2_SO_4_ + 0.5 M K_2_SO_4_ | This work |
| Co_3_O_4_/TNBs// bioanode | 0.076 | 0.089 | 4.6 | bias-free | 10 | 0.5 M Na2SO4 | ^[10]^ |
| BiOI-Bi //TiO_2_ | 180.2 | 1.14 | 96 | 1.2 | 12 | 0.5 M KHCO_3_ | ^[11]^ |
| RuCP/ carbon//IrOx/3jn-SiG | 25.1 | 0.025 | 78 | bias-free | 3 | 0.1 M KPi | ^[12]^ |
| Cu-S\|\|Si-based //NiFeZnOx | 193 | 17 | 59.7 | 2.5 | 10 | 0.1 M KHCO_3_ | ^[13]^ |
| RuCP@C//c-Si\|IrOx | 120 | 6.44 | 80 | 1.85 | - | 0.4 M KPi | ^[14]^ |
| RuCP@Ti //c-Si/IrOx | 122 | 0.9 | 96 | 1.65 | 10 | 0.4 M KPi | ^[15]^ |
| CIFDH/TiO_2_/CFO//FeOOH/BiVO_4_ | 0.098 | 0.4 | 33.5 | 0.4 | 12 | 0.4 M KPi | ^[16]^ |
| IO-TiO_2_\|W-FDH//IO-TiO2\|dpp\|POs-PSII | 2.22 | 0.092 | 70 | 0.6 | 1 | 0.1 M NaHCO_3_+0.05M KCl | ^[17]^ |
| Sn-Bi_2_O_3_ // 3-J(InGaP/GaAs/Ge)\|Ni/RuOx | 167.9 | 10 | 88 | bias-free | 100 | 0.1 M KHCO_3_ | ^[18]^ |
| Bi/GaN/Si // NiOOH/α-Fe_2_O_3_ | 23.3 | 1.25 | 90 | 1.1 | 80 | 0.1 M KHCO_3_ | ^[19]^ |
| PVK\|IO-TiO_2_\|FDH//BiVO | 7.1 | 0.7 | 83 | bias-free | 10 | 0.1M NaHCO_3_ +0.05M CsCl | ^[20]^ |
| p-type Co_3_O_4_ nanorod //bioanode | 7.83 | 4 | 10.5 | bias-free | 10 | 0.1 M Na_2_SO_4_ | ^[21]^ |
| In_0.4_Bi_0.6_-coated perovskite // Pt | 92.35 | 5.5 | 90 | 1.6 | 1.5 | KHCO3 | ^[22]^ |
| 3D TiN-ClFDH // BiVO_4_/perovskite | 2.26 | 0.082 | 82.7 | 0.9 | 24 | sodium phosphate buffer aqueous | ^[23]^ |
| Ag//WO_3_/BiVO_4_ | 87.78 | 5 | 94.1 | 1.7 | 3.8 | [NH2C3MIm][Br] solution | ^[24]^ |
| Pd/C-coated Ti // GaAs/InGaP/TiO_2_/Ni | 143 | 8.7 | 95 | 2.04 | 3 | 0.1M KHCO_3_ | ^[25]^ |

**3. Reference**

[1] J. Shao, H. Jing, P. Wei, X. Fu, L. Pang, Y. Song, K. Ye, M. Li, L. Jiang, J. Ma, R. Li, R. Si, Z. Peng, G. Wang, J. Xiao, *Nat. Energy* **2023**, *8*, 1273.

[2] L. Shang, X. Lv, L. Zhong, S. Li, G. Zheng, *Small Methods.* **2022**, *6*, 2101334.

[3] W. Gao, Y. Xu, L. Fu, X. Chang, B. Xu, *Nat. Catal.* **2023**, *6*, 885.

[4] E. P. Delmo, Y. Wang, Y. Song, S. Zhu, H. Zhang, H. Xu, T. Li, J. Jang, Y. Kwon, Y. Wang, M. Shao, *J. Am. Chem. Soc.* **2024**, *146*, 1935.

[5] G. Kresse, J. Furthmüller, *Phys. Rev. B* **1996**, *54*, 11169.

[6] G. Kresse, J. Furthmüller, *Comput. Mater. Sci.* **1996**, *6*, 15.

[7] M. Ernzerhof, G. E. Scuseria, *J. Chem. Phys.* **1999**, *110*, 5029.

[8] S. Grimme, *J. Comput. Chem.* **2006**, *27*, 1787.

[9] P. E. Blöchl, *Phys. Rev. B* **1994**, *50*, 17953.

[10] J. Wu, G. Zhao, S. Yin, S. Tian, J. Qi, S. Wang, D. Li, Y. Feng, *Appl. Catal., B* **2025**, *361*, 124630.

[11] J. Zhao, L. Xue, Z. Niu, L. Huang, Y. Hou, Z. Zhang, R. Yuan, Z. Ding, X. Fu, X. Lu, J. Long, *J. Power Sources* **2021**, *512*, 230532.

[12] T. Morikawa, S. Sato, K. Sekizawa, T. M. Suzuki, T. Arai, *Acc. Chem. Res.* **2022**, *55*, 933.

[13] C. Ampelli, D. Giusi, M. Miceli, T. Merdzhanova, V. Smirnov, U. Chime, O. Astakhov, A. J. Martín, F. L. P. Veenstra, F. A. G. Pineda, J. González-Cobos, M. García-Tecedor, S. Giménez, W. Jaegermann, G. Centi, J. Pérez-Ramírez, J. R. Galán-Mascarós, S. Perathoner, *Energy Environ. Sci.* **2023**, *16*, 1644.

[14] N. Kato, S. Mizuno, M. Shiozawa, N. Nojiri, Y. Kawai, K. Fukumoto, T. Morikawa, Y. Takeda, *Joule* **2021**, *5*, 687.

[15] N. Kato, Y. Takeda, Y. Kawai, N. Nojiri, M. Shiozawa, S. Mizuno, K. I. Yamanaka, T. Morikawa, T. Hamaguchi, *ACS Sustainable Chem. Eng.* **2021**, *9*, 16031.

[16] S. K. Kuk, J. Jang, J. Kim, Y. Lee, Y. S. Kim, B. Koo, Y. W. Lee, J. W. Ko, B. Shin, J. K. Lee, C. B. Park, *ChemSusChem* **2020**, *13*, 2940.

[17] K. P. Sokol, W. E. Robinson, A. R. Oliveira, J. Warnan, M. M. Nowaczyk, A. Ruff, I. A. C. Pereira, E. Reisner, *J. Am. Chem. Soc.* **2018**, *140*, 16418.

[18] B. Khan, M. B. Faheem, K. Peramaiah, J. Nie, H. Huang, Z. Li, C. Liu, K. W. Huang, J. H. He, *Nat. Commun.* **2024**, *15*, 6990.

[19] Y. Pan, H. Zhang, B. Zhang, F. Gong, J. Feng, H. Huang, S. Vanka, R. Fan, Q. Cao, M. Shen, Z. Li, Z. Zou, R. Xiao, S. Chu, *Nat. Commun.* **2023**, *14*, 1013.

[20] E. Edwardes Moore, V. Andrei, A. R. Oliveira, A. M. Coito, I. A. C. Pereira, E. Reisner, *Angew. Chem. Int. Ed.* **2021**, *60*, 26303.

[21] J. Wu, X. Han, D. Li, B. E. Logan, J. Liu, Z. Zhang, Y. Feng, *Appl. Catal., B* **2020**, *276*, 119102.

[22] J. Chen, J. Yin, X. Zheng, H. Ait Ahsaine, Y. Zhou, C. Dong, O. F. Mohammed, K. Takanabe, O. M. Bakr, *ACS Energy Lett.* **2019**, *4*, 1279.

[23] S. K. Kuk, Y. Ham, K. Gopinath, P. Boonmongkolras, Y. Lee, Y. W. Lee, S. Kondaveeti, C. Ahn, B. Shin, J. K. Lee, S. Jeon, C. B. Park, *Adv. Energy Mater.* **2019**, *9*, 1900029.

[24] W. Lu, B. Jia, B. Cui, Y. Zhang, K. Yao, Y. Zhao, J. Wang, *Angew. Chem. Int. Ed.* **2017**, *56*, 11851.

[25] X. Zhou, R. Liu, K. Sun, Y. Chen, E. Verlage, S. A. Francis, N. S. Lewis, C. Xiang, *ACS Energy Lett.* **2016**, *1*, 764.
